# The Radiosensitizing Effect of Zinc Oxide Nanoparticles in Sub-Cytotoxic Dosing Is Associated with Oxidative Stress In Vitro

**DOI:** 10.3390/ma12244062

**Published:** 2019-12-05

**Authors:** Till Jasper Meyer, Agmal Scherzad, Helena Moratin, Thomas Eckert Gehrke, Julian Killisperger, Rudolf Hagen, Gisela Wohlleben, Bülent Polat, Sofia Dembski, Norbert Kleinsasser, Stephan Hackenberg

**Affiliations:** 1 Department of Oto-Rhino-Laryngology, Plastic, Aesthetic and Reconstructive Head and Neck Surgery, University Hospital Wuerzburg, Josef-Schneider-Str. 11, D-97080 Würzburg, Germany; scherzad@ukw.de (A.S.); moratin_h@ukw.de (H.M.); gehrke_t@ukw.de (T.E.G.); julian.killisperger@stud-mail.uni-wuerzburg.de (J.K.); hagen_r@ukw.de (R.H.); hackenberg_s@ukw.de (S.H.); 2 Department of Radiation Oncology, University Hospital of Wuerzburg, Josef-Schneider-Str. 11, D-97080 Würzburg, Germany; Wohlleben_G@ukw.de (G.W.); polat_b@ukw.de (B.P.); 3 Fraunhofer Institute for Silicate Research ISC, Neunerplatz 2, D-97082 Würzburg, Germany; sofia.dembski@isc.fraunhofer.de; 4 Department of Tissue Engineering and Regenerative Medicine (TERM), University Clinic Würzburg, Röntgenring 11, 97070 Würzburg, Germany; 5 Department of Oto-Rhino-Laryngology, Head and Neck Surgery, Kepler University Hospital, A-4021 Linz, Austria; norbert.kleinsasser@kepleruniklinikum.at

**Keywords:** zinc oxide nanoparticles, irradiation, oxidative DNA damage, head and neck squamous cell carcinoma

## Abstract

Radioresistance is an important cause of head and neck cancer therapy failure. Zinc oxide nanoparticles (ZnO-NP) mediate tumor-selective toxic effects. The aim of this study was to evaluate the potential for radiosensitization of ZnO-NP. The dose-dependent cytotoxicity of ZnO-NP_20 nm_ and ZnO-NP_100 nm_ was investigated in FaDu and primary fibroblasts (FB) by an MTT assay. The clonogenic survival assay was used to evaluate the effects of ZnO-NP alone and in combination with irradiation on FB and FaDu. A formamidopyrimidine-DNA glycosylase (FPG)-modified single-cell microgel electrophoresis (comet) assay was applied to detect oxidative DNA damage in FB as a function of ZnO-NP and irradiation exposure. A significantly increased cytotoxicity after FaDu exposure to ZnO-NP_20 nm_ or ZnO-NP_100 nm_ was observed in a concentration of 10 µg/mL or 1 µg/mL respectively in 30 µg/mL of ZnO-NP_20 nm_ or 20 µg/mL of ZnO-NP_100 nm_ in FB. The addition of 1, 5, or 10 µg/mL ZnO-NP_20 nm_ or ZnO-NP_100 nm_ significantly reduced the clonogenic survival of FaDu after irradiation. The sub-cytotoxic dosage of ZnO-NP_100 nm_ increased the oxidative DNA damage compared to the irradiated control. This effect was not significant for ZnO-NP_20 nm_. ZnO-NP showed radiosensitizing properties in the sub-cytotoxic dosage. At least for the ZnO-NP_100 nm_, an increased level of oxidative stress is a possible mechanism of the radiosensitizing effect.

## 1. Introduction

With an estimated 65,000 new cases in 2019, head and neck squamous cell carcinoma (HNSCC) is the seventh most common cancer in the United States [1]. Despite all innovations in diagnostics and therapy, the five-year overall survival of HNSCC patients remains poor, at around 50% [2].

Surgical intervention and/or radiation is the backbone of HNSCC patients’ therapy. Although the precision of radiation has been increased by introducing technologies such as intensity-modulated radiotherapy, damage to non-tumor tissues near the tumor is unavoidable [3]. In order to increase the antitumor effect of the radiation and to focus the effect of the radiation on the tumor volume, radiosensitizing agents such as cisplatin-chemotherapy are used in special clinical risk situations like primary radiation therapy, close or positive tumor margins, perineural invasion, or extranodal lymph node spread [4,5].

Nanoparticles (NP) are defined by a particle size of less than 100 nm. Due to their ratio of surface area to particle mass, NP have special physico-chemical properties [6]. Zinc oxide (ZnO)-NP is moving into the focus of medical research due to its potential to trigger tumor-selective cell death [7] and as a cancer-inhibiting drug carrier [8]. In addition, different groups could show a biological distribution characterized by greater accumulation of ZnO-NP in tumors as compared to healthy tissue [9]. By UV-light activation, ZnO-NP induce a photocatalytic cancer cell death [10,11]. However, the use of photodynamic therapy is limited to tissue surfaces mainly because of the low tissue penetration of light. A photosensitizer activated by X-rays would, therefore, be desirable [12].

The exact mechanism of ZnO-NP’s mediation in tumor-cell toxicity is unknown. In this context, ZnO-NP-catalyzed production of reactive oxygen species (ROS) is most frequently discussed [7,13], which leads to increased intracellular oxidative stress. Subsequent induction of apoptosis, cell cycle changes [14], and pro-inflammatory processes [15] is frequently observed.

Considering the tumor-selective toxic effects and the concentration of ZnO-NP in tumor tissue, ZnO-NP have a theoretical radiosensitive potential.

The aim of this study was to investigate the radiosensitizing properties of the subtoxic ZnO-NP dosage in HNSCC cells. Furthermore, the influence of ZnO-NP alone and in combination with radiation on oxidative DNA damage was assessed.

## 2. Materials and Methods

### 2.1. Preparation of ZnO-NP Suspension

ZnO-NP of 20 nm size (ZnO-NP_20 nm_) (20 nm, specific surface area 90 m^2^/g) were obtained from MKnano (Mississauga, Ontario, Canada) and ZnO-NP of 100 nm size (ZnO-NP_100 nm_) (<100 nm, specific surface area 10–25 m^2^/g) were obtained from Sigma-Aldrich (St. Louis, Missouri, MI, USA).

In order to achieve a good particle dispersion, the NP solution was prepared according to the protocol of Bihara et al. [16]. A total of 20 mg ZnO-NP was dissolved in 1.740 mL distilled aqua and subsequently sonicated with 4.2 × 10^5^ kJ/m^3^ (Sonopuls HD 60, Bandelin, Berlin, Germany) for 120 s in the continuous mode.

To stabilize the suspension, 60 µL of 1.5 mg/mL bovine serum albumin (BSA) was added. By adding 200 µL of 10-fold concentrated phosphate-buffered saline (PBS) with physiological pH 7.4, the necessary salt concentrations were achieved. The stock suspension with PBS was further diluted to achieve various concentrations of ZnO-NP for the experiments.

### 2.2. Characterization of ZnO Nanoparticles

The ZnO-NP were previously characterized by our group [17,18]. Transmission electron microscopy (TEM) (Zeiss transmission electron microscope EM 900, Carl Zeiss, Oberkochen, Germany) was used to assess particle size distribution and morphology at the Division of Electron Microscopy at the Biocenter of University of Würzburg. The size distribution of NP agglomerates and the zeta potential were determined by dynamic light scattering, after preparation of the ZnO-NP suspension as described above and dilution with cell culture medium to a concentration of 10 µg/mL (Malvern Instruments Ltd., Herrenberg, Germany).

### 2.3. Cell Culture

For the experiments, the HNSCC cell line FaDu, isolated from a hypopharyngeal carcinoma, was used [19]. The cells were cultured under standard conditions (37 °C, 5% CO_2_) in RPMI-1640 medium (Biochrom Ltd., Cambridge, UK) with a supplement of 10% fetal calf serum (FCS) (Linaris Blue Wertheim-Bettingen, Germany), 100 μg/mL streptomycin, 100 U/mL penicillin, 1% sodium pyruvate (100 mM; Biochrom Ltd.), and 1% non-essential amino acids (100-fold concentration; Biochrom Ltd.). The medium was changed every other day. Cells were passaged with trypsin (0.25% trypsin; Gibco; Thermo Fisher Scientific, Waltham, USA) after reaching a confluence of 70–80%.

The fibroblasts were isolated from skin samples of patients who received elective neck surgery. All patients gave informed consent. This study was approved by the Ethics Committee of the University of Würzburg (approval no. 116/17). Fibroblasts were isolated from the skin according to the protocol described by Vangipuram et al. [20] and as previously described by our group [21]. Briefly, the fat residuals were removed from the skin sample and then cut into 2–3 mm pieces. The skin pieces were placed on a petri dish. After 60 min of waiting for attachment of the tissue pieces to occur, Dulbecco’s Modified Eagle Medium (DMEM, Invitrogen, Thermo Fisher Scientific) supplemented with 10% FCS, 100 μg/mL streptomycin, and 100 U/mL penicillin were added, taking care to avoid flushing away of the skin pieces. The medium was changed every other day. Cell passaging was done with trypsin (0.25% trypsin; Gibco; Thermo Fisher Scientific, Waltham, USA); a confluence of 70–80% was reached. The growth of the fibroblasts at the bottom of the petri dish was monitored by microscopy (DM IL LED, Leica, Wetzlar, Germany).

### 2.4. MTT Cytotoxicity Assay

The cell viability after exposure to ZnO-NP in different concentrations was evaluated by the MTT [3-(4,5-dimethylthiazol-2-yl)-2.5-diphenyl tetrazolium bromide] colorimetric staining method [22]. The cells were incubated with 1 mg/mL of MTT (Sigma-Aldrich) dissolved in the medium for 4 h under cell culture conditions. Isopropanol was added to solubilize the crystal formations for one hour. The photometric measurement of the color conversion was performed at 570 nm wavelength with a multiplate reader (Titertek Multiskan PLUS MK II, Labsystems, Helsinki, Finland). The half maximal inhibitory concentrations (IC_50_) were calculated after performing first a logarithm transformation of the ZnO-NP concentration values, and second, nonlinear regression by the use of the Prism software (GraphPad software, San Diego, CA, USA).

### 2.5. Cell Cycle Analysis

After incubation of FaDu and fibroblasts (FB) with 0, 1, 5, and 10 µg/mL ZnO-NP_20 nm_ and ZnO-NP_100 nm_ for 24 h, the cells were fixed with 70% ethanol at 4 °C in the dark for 2 h. Next, the cells were exposed to 500 µL PI/RNase staining buffer (Becton–Dickinson, Heidelberg, Germany) at 4 °C in the dark for 15 min and subsequently analyzed by flow cytometry (FACScanto, Becton–Dickinson).

### 2.6. Irradiation

X-irradiation was performed using a 6 MV Siemens linear accelerator (Siemens, Concord, CA, USA) with a dose rate of 2 Gy/min.

### 2.7. Clonogenic Survival Assay

The influence of ZnO-NP on the survival of tumor cells after irradiation was investigated by a clonogenic survival assay. After seeding 2 × 10^3^ FaDu cells to a six-well dish, ZnO-NP treatment was performed for 24 h. In addition, X-ray irradiation was performed with 5 Gy. The irradiated cells were incubated in a humidified atmosphere (5% CO_2_, 37 °C) for 10 days. Cell fixation was performed with methanol and acetic acid in a ratio of 3:1 and stained with 0.1% crystal violet. Colonies with at least 50 cells were counted.

### 2.8. FPG-Modified Single-Cell Microgel Electrophoresis (Comet) Assay

In order to detect oxidized bases, alkali labile sites, and incomplete excision repair sites as markers for oxidative DNA damage, the formamidopyrimidine-DNA glycosylase (FPG)-modified comet assay was used. Therefore, the alkali version of the comet assay was modified by the additional use of the FPG protein [23,24]. The classical comet assay was performed as described by our group earlier [25].

The cells were seeded into a 6-well plate (2 × 105 cells/well). After overnight cultivation, they were exposed to 0 and 1 µg/mL ZnO-NP_20 nm_ and ZnO-NP_100 nm_ for 24 h. As a positive control, another sample was incubated with 400 mM methyl methanesulphonate (MMS, Sigma-Aldrich) for 1 h. Immediately after irradiation with 0 and 5 Gy, the cells were washed with PBS, trypsinized, and dissolved in the medium. All further steps were performed on ice to reduce further DNA damage. A cell pellet was created by centrifugation at 1500 rpm for 5 min. Subsequently, the cell pellet was resuspended in 0.5% low melting agarose (Sigma-Aldrich) and transferred to a slide. The specimen slides (Langenbrinck, Emmendingen, Germany) were coated with 1.5% normal melting agarose (Roth, Karlsruhe, Germany). The cells were incubated in the lysis buffer (100 mM Na2EDTA pH 10, 10% DMSO, 1% Triton-X, 2.5 M NaCl, 10 mM Tris) at 4 °C for 2h. Afterward, 100 µL lysis buffer containing 20% BSA and ± 0.03% FPG-enzyme (New England Biolabs, Frankfurt, Germany) was added at 37 °C for 30 min. After transferring the slides to the gel electrophoresis chamber (Renner, Darmstadt, Germany), alkaline buffer (5 mM NaOH and 200 mM Na2EDTA) with a pH > 13 was added. Gel electrophoresis was performed for 20 min (25 V, 300 mA). Subsequently, the cells were neutralized to pH 7.5 and stained with 20 µL GelRed (Biotium, Fremont, CA, USA).

Further specimen slide evaluations were performed with the DMLB fluorescence microscope (Leica Microsystems, Wetzlar, Germany) and the image analysis system (Komet 5.5, Kinetic Imaging, Liverpool, UK). From each slide, 50 cells were examined with a focus on the following parameters: Percentage of DNA in tail, tail length, and Olive tail moment (OTM), which is the product of the percentage of DNA in the comet tail and the median migration distance [26].

For assessment of genotoxicity attributed to oxidative stress, the Δ fpg OTM was calculated by subtracting the OTM of the fpg-negative comet assay from the OTM of the fpg-positive comet assay. The calculation of the Δ fpg tail DNA was processed in the same way.

### 2.9. Trypan Blue Exclusion Test

To evaluate the cell viability after incubation with ZnO-NP and irradiation, the trypan blue exclusion test was performed in a Neubauer Chamber. A total of 16 counting fields were examined to compute the percentage of viable cells.

### 2.10. Statistical Analysis

One-way ANOVA and multiple comparison testing were applied to determine statistically relevant differences in the mean values in comparison to the negative control in the MTT assay, clonogenic survival assay, and FPG-comet assay. A *p*-value of < 0.05 was set as significant and marked with an asterisk.

## 3. Results

### 3.1. ZnO-NP Characterization

ZnO-NP_20 nm_ had a mean diameter of 20–30 nm, measured with TEM. The mean diameter of the particle aggregates assessed in the culture medium was 67.1 nm, and the zeta potential was −11.2 mV.

ZnO-NP_100 nm_ showed a mean diameter of 45–55 nm in TEM. The mean diameter of the particles aggregated and assessed in the culture medium was 120.68 nm. The zeta potential was −11.2 mV and the polydispersity index was 0.136.

### 3.2. ZnO-NP-Mediated Cytotoxicity

#### 3.2.1. ZnO-NP-Mediated Cytotoxicity in FaDu

**Figure 1:**
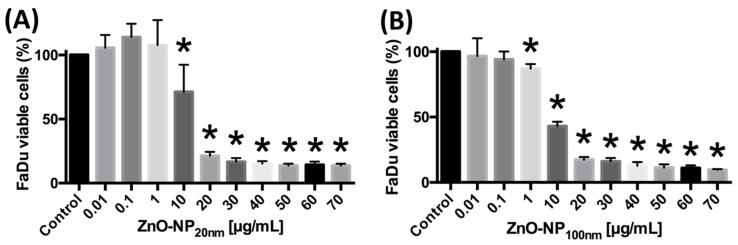
FaDu viability assessed by MTT assay in dependence of (**A**) ZnO-NP_20 nm_ and (**B**) ZnO-NP_100 nm_ (zinc oxide nanoparticle) solutions of various concentrations. The figure shows the results of three independent experiments. Significant differences arising from comparison with the untreated control cells are marked with *.

The cell vitality of FaDu was evaluated after exposure to ZnO-NP_20 nm_ and ZnO-NP_100 nm_ in different concentrations for 24 h by the MTT assay. A significant reduction of cell viability was observed after exposure to ZnO-NP_20 nm_ at concentrations of 10 µg/mL and higher (Figure 1). After incubation with ZnO-NP_100 nm_, a significant decrease in cell viability was detected at concentrations of 1 µg/mL and higher (Figure 1). The IC_50_ values were 13.8 µg/mL for ZnO-NP_20 nm_ and 6.4 µg/mL for ZnO-NP_100 nm_ in FaDu.

#### 3.2.2. ZnO-NP-Mediated Cytotoxicity in FB

**Figure 2:**
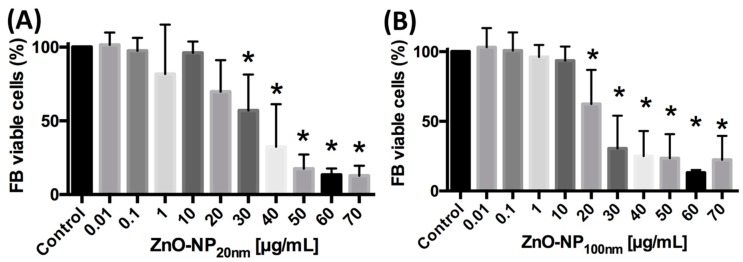
Fibroblast (FB) viability after treatment with (**A**) ZnO-NP_20 nm_ and (**B**) ZnO-NP_100 nm_, measured by MTT assay, is significantly reduced in higher ZnO-NP concentrations as compared to FaDu (Figure 1). The figure shows the results of testing FB of five different donors. Significant differences in comparison with untreated control cells are marked with *.

Cytotoxicity with respect to a significant reduction of FB viability was observed after exposure to ZnO-NP_20 nm_ at concentrations of 30 µg/mL and higher (Figure 2). The vitality of the FB after incubation with ZnO-NP_100 nm_ at concentrations of 20 µg/mL and higher was significantly decreased (Figure 2). The IC_50_ values were 30.4 µg/mL for ZnO-NP_20 nm_ and 24.6 µg/mL for ZnO-NP_100 nm_ in FB.

### 3.3. Influence of ZnO-NP on the Cell Cycle Distribution in FaDu and FB

**Figure 3:**
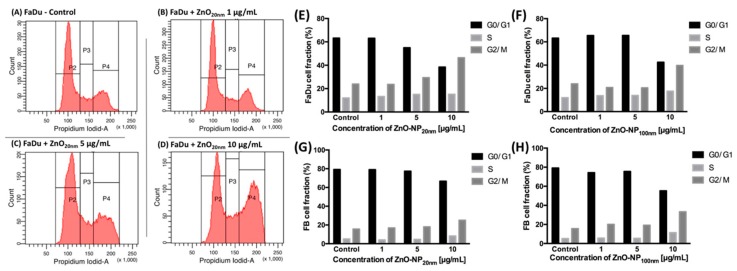
Cell cycle distribution of FaDu and FB in ZnO-NP_20 nm_ and ZnO-NP_100 nm_ solutions of various concentrations measured by propidium iodide flow cytometry. (**A**–**D**) show one exemplary analysis after incubation of FaDu cells with different concentrations of ZnO-NP_20 nm_. Due to exposure to 10 µg/mL ZnO-NP, the cell cycle distribution shifted to a higher fraction of the G2/M-phases in (**E**) + (**F**) FaDu and (**G**) + (**H**) FB.

The influence of ZnO-NP exposure to FaDu and FB on cell cycle distribution was assessed by propidium iodide flow cytometry. Incubation with ZnO-NP_20 nm_ and ZnO-NP_100 nm_ at concentrations of 10 µg/mL for 24 h led to a cell cycle shift to the G2/M-phases in FaDu and FB (Figure 3). Subsequently, the number of cells with a low staining intensity with propidium iodide decreased under the influence of 10 µg/mL of ZnO-NP_20 nm_ and ZnO-NP_100 nm_ (Figure 3).

### 3.4. Colonial Cell Survival in Relation to ZnO-NP Concentration and Irradiation

**Figure 4:**
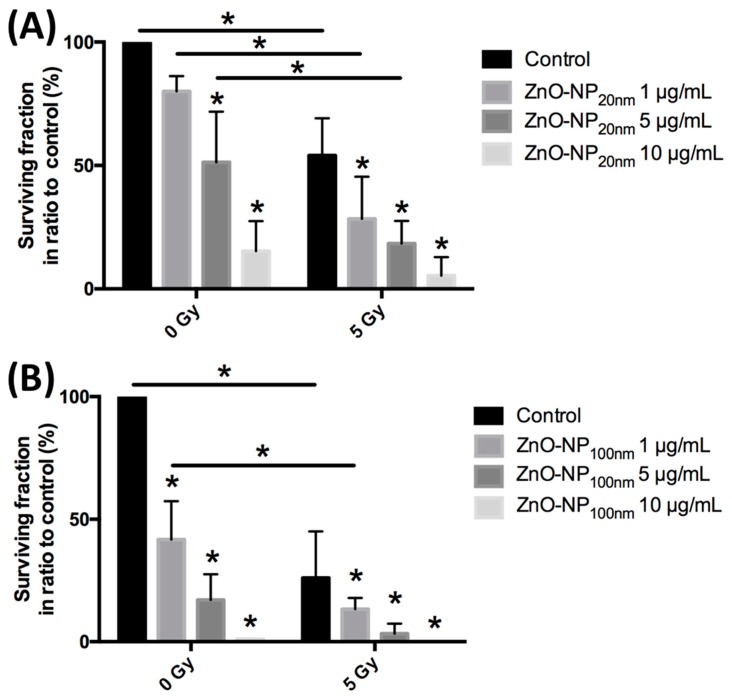
Clonogenic survival of FaDu after exposure to 0 Gy or 5 Gy irradiation and to (**A**) ZnO-NP_20 nm_ and (**B**) ZnO-NP_100 nm_. All statistically significant differences in comparison to the control within the 0 Gy and 5 Gy groups are marked with an * over the column. Furthermore, all statistically significant differences after treatment with the same concentration of ZnO-NP are marked with an * on the girder. The data represent the mean ± standard deviation from three independent experiments.

After irradiation with 5 Gy, clonogenic survival was significantly reduced in all investigated constellations compared to the non-irradiated FaDu cells (Figure 4). The addition of ZnO-NP_20 nm_ or ZnO-NP_100 nm_ at concentrations of 1, 5, and 10 µg/mL significantly reduced the survival of the cells within the non-irradiated and the irradiated groups, with the exception of non-irradiated FaDu exposed to ZnO-NP_20 nm_ at 1 µg/mL concentration (Figure 4).

### 3.5. Cytotoxicity Assessment by the Trypan Blue Exclusion Test

**Figure 5:**
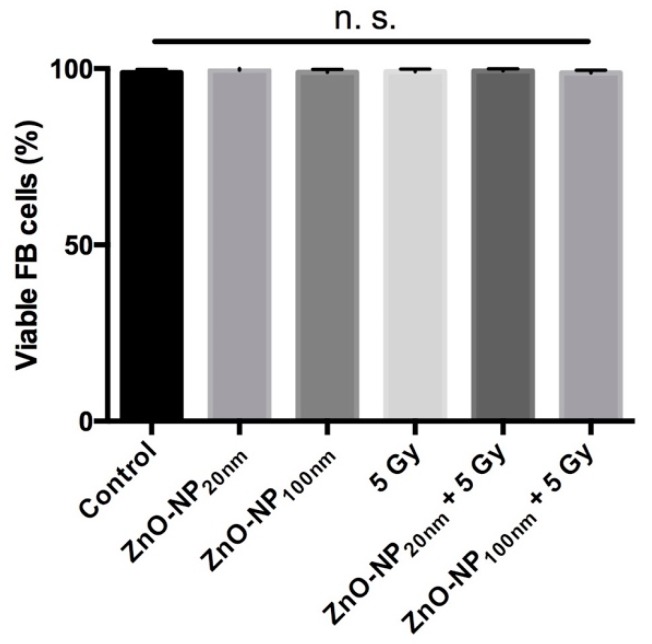
Viability of FB cells after treatment with ZnO-NP and/or radiation. There is no significant reduction of cell viability revealed by the trypan blue test.

After exposure to ZnO-NP and irradiation and prior to the comet assay, cytotoxicity was excluded by the trypan blue test. It did not reveal a significant influence on FB viability for the chosen ZnO-NP concentrations and the irradiation doses (Figure 5).

### 3.6. DNA Damage and Changes of Oxidative Stress after Exposure to ZnO-NP and Irradiation

#### 3.6.1. DNA Damage Immediately after Exposure to ZnO-NP and Irradiation

**Figure 6:**
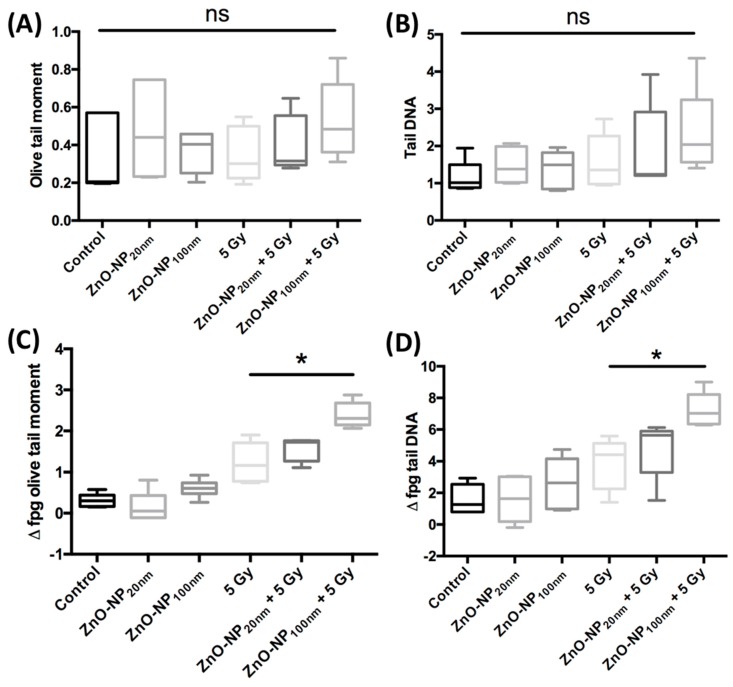
DNA damage and oxidative stress after FB exposure to ZnO-NP 1 µg/mL and ±5 Gy irradiation evaluated by conventional comet assay (**A**,**B**) and after addition of the formamidopyrimidine-DNA glycosylase (FPG)-enzyme (**C**,**D**). There is no significant difference in DNA damage measured by (**A**) OTM and (**B**) percentage of tail DNA in the conventional comet assay. As compared to the irradiated cells without incubation with ZnO-NP, those exposed to ZnO-NP_100 nm_ show greater oxidative stress, as seen in (**C**) Δ fpg OTM and (**D**) Δ fpg tail DNA. The data represents experiments with five individual FB cells.

There was no significant change in DNA damage in FB as evaluated by the OTM and the percentage of tail DNA in the conventional comet assay without treatment or after treatment with 1 µg/mL ZnO-NP and 5 Gy irradiation (Figure 6).

#### 3.6.2. Changes of Oxidative Stress Immediately after Exposure to ZnO-NP and Irradiation

The induction of oxidative stress after exposure to ZnO-NP alone and in combination with irradiation was investigated with the comet assay after the addition of the FPG-enzyme. Oxidative stress in terms of Δ fpg OTM was significantly increased after irradiation as compared to non-irradiated cells. After exposure to ZnO-NP_100 nm_ and irradiation, Δ fpg OTM and Δ fpg tail DNA were significantly increased as compared to cells that were only irradiated (Figure 6). Δ fpg OTM and Δ fpg tail DNA were not significantly raised after incubation with ZnO-NP_20 nm_ and irradiation compared to irradiation alone (Figure 6).

## 4. Discussion

Selective tumor-cell death mediated by ZnO-NP is discussed as a promising characteristic of ZnO-NP for its use as an anti-cancer drug [7,27,28]. We observed the cytotoxicity of ZnO-NP in FaDu cells and FB. However, toxic concentrations of ZnO-NP in FB were significantly higher as compared to concentrations in FaDu, indicating a higher susceptibility of the malignant FaDu cells to NP-mediated cell death. High tolerance of non-malignant cells for oxidative stress as reported by He et al. could be one possible reason for the observed differences in ZnO-NP-mediated cytotoxic effects in FaDu and FB [29]. In particular, the authors described a high expression of antioxidant enzymes such as manganese superoxide dismutase in human endothelial progenitor cells [29].

Although the exact mechanism of ZnO-NP-mediated toxicity for tumor cells is unknown, several mechanisms have been discussed. Various groups have described the ZnO-NP-dependent intracellular production of reactive oxygen species (ROS) [7,13]. Increased concentrations of intracellular ROS induce a metabolic status of oxidative stress. Oxidative stress is associated with apoptosis, cell cycle alterations [14], pro-inflammatory processes [15], and DNA damage [30,31].

Another possible mechanism of ZnO-NP-mediated toxicity could be the high content of dissolved Zn^2+^ cations [30,32]. In central areas of solid tumor formations, insufficient oxygen supply often leads to low pH values. In theory, this can promote the dissolution of divalent Zn^2+^ ions from ZnO-NP. This may possibly explain the higher tumor-specific toxicity of ZnO-NP. But due to the controlled normoxic and pH conditions in the performed cell culture experiments, the effects of dissolved Zn^2+^ cations in in vitro experiments are expected to be low.

The effectiveness of irradiation depends on the specific dosage and is mainly limited by the damage that irradiation causes to the healthy tissue surrounding the tumor. Radiosensitizing drugs that focus the toxic effects of radiation on tumor tissue and protect the surrounding non-tumor tissue as much as possible would be desirable. The tumor-cell-selective toxic effects of ZnO-NP could prove ZnO-NP to be a potential radiosensitizer. In our study, ZnO-NP, even at the low dose of 1 µg/mL, significantly reduced the clonogenic survival of FaDu cells after irradiation. In contrast to our results, Sadjadpour et al. observed no increased cytotoxic effects after combined irradiation and ZnO-NP treatment in breast cancer cells [33]. However, the low radiation dose (1 Gy) and the evaluation of cytotoxicity by MTT assay, which does not reliably detect radiotherapy-mediated toxicity, influenced the results. In contrast, other groups described a ZnO-NP-dependent sensitivity to irradiation. For example, Zangeneh et al. observed increased cytotoxic and genotoxic effects of gadolinium-doped ZnO-NP on irradiated lung cancer cells at megavoltage radiation energies [34]. Generalov et al. could show a ZnO-NP-mediated sensitization for irradiation in the prostate carcinoma cell lines LNCaP and Du145 [35].

Despite several reports on the radiosensitivity mediated by ZnO-NP, the exact mechanisms of this action are still unclear. One theory assumes that ZnO-NP mediates by causing a higher rate of oxidation ROS generation, and that increases the ionizing effect of the radiation [36,37]. In our study, the oxidative DNA damage measured by Δ fpg OTM and Δ fpg tail DNA was not significantly increased after exposure to ZnO-NP_20 nm_ and ZnO-NP_100 nm_ in the low dosage of 1 µg/mL. However, there was a significant increase in Δ fpg OTM and Δ fpg tail DNA after exposure to ZnO-NP_100 nm_ and irradiation, in comparison with irradiation of cells only. These data suggest that ZnO-NP-mediated radiosensitization may be associated with oxidative stress, at least for ZnO-NP_100 nm_. However, these results were not significant for ZnO-NP_20 nm_. Our observation of higher cytotoxic and oxidative DNA damage potential of ZnO-NP_100 nm_ compared to that of the ZnO-NP_20 nm_ is surprising. A trend of size-dependent higher toxic potential of NP towards finer NP has been controversially discussed [38,39,40].

Tumor hypoxia is one of the main challenges in radioresistance in HNSCC [41]. The causes are rapid tumor growth, lack of compensatory angiogenesis, and metabolic changes in the tumor [42]. To the best of our knowledge, there is no report on the radiosensitizing effects of ZnO-NP under hypoxic conditions. For ROS generation in the reductive pathway, the presence of oxygen is necessary. Irradiation of ZnO-NP-exposed biological material leads to ROS generation by transferring ZnO-released electrons to oxygen [37]. For a comprehensive evaluation of the radiosensitization potential of ZnO-NP, the radiosensitive potential of ZnO-NP under hypoxic conditions should be evaluated concretely.

We observed an influence of ZnO-NP on the cell cycle in terms of G2/M arrest at high ZnO-NP concentrations. This ZnO-NP-mediated effect on the cell cycle was previously described by various other groups [17,43]. The increased radiation sensitivity in our experiments, even at low ZnO-NP concentrations, questions the ZnO-NP-mediated G2/M arrest as the main mechanism that causes radiation sensitivity.

In summary, our data suggest that ZnO-NP has a radiosensitization potential in FaDu. At least in the case of ZnO-NP_100 nm_, increased oxidative stress seems to be an important mechanism for the ZnO-NP-mediated radiation-sensitization effect.
